# The J shaped association of age at menarche and cardiovascular events: systematic review and meta-analysis

**DOI:** 10.1038/s41598-024-53011-5

**Published:** 2024-02-01

**Authors:** Samira Behboudi-Gandevan, Cathrine Fredriksen Moe, Ingunn Skjesol, Ellen Christin Arntzen, Razieh Bidhendi-Yarandi

**Affiliations:** 1 Faculty of Nursing and Health Sciences, Nord University, Post Box: 1490, 8049 Bodø, Norway; 2 Faculty of Nursing and Health Sciences, Nord University, Namsos, Norway; 3 Department of Biostatistics and Epidemiology, School of Social Health, University of Social Welfare and Rehabilitation Sciences, Tehran, Iran; 4 Social Determinants of Health Research Center, University of Social Welfare and Rehabilitation Sciences, Tehran, Iran

**Keywords:** Risk factors, Diseases, Cardiovascular diseases, Reproductive disorders

## Abstract

This study aimed to evaluate the association between age at menarche and cardiovascular (CV) events through a systematic review and meta-analysis of observational studies. A comprehensive literature search covering studies published from January 1, 2000, to October 31, 2023, was conducted in PubMed, MEDLINE, Embase, and Scopus. Twenty-nine observational studies involving 4,931,160 adult women aged 18 years or older were included. The meta-analysis revealed a J-shaped association between age at menarche and CV events. Individuals with menarche at 12–13 years exhibited the lowest risk, while those with younger (≤ 11 years) or older ages (14–15 years and ≥ 16 years) showed an increased risk. Notably, individuals with age at menarche of 16 years and older had the highest risk of CV events. The pooled odds of CV mortality in age at menarche categories 14–15 years and ≥ 16 years were 37% (OR: 1.37, 95% CI 1.14–1.64, I^2^: 76.9%) and 64% (OR: 1.64, 95% CI 1.20–2.24, I^2^: 87%) higher than referent age at menarche 12–13 years. No statistically significant difference was found in CV mortality risk between individuals with age at menarche ≤ 11 years and those with age at menarche 12–13 years. The ORs for coronary heart disease were significantly higher for age at menarche ≥ 16 years (35% increase), while no significant difference was found for age at menarche ≤ 11 years or 14–15 years compared to age at menarche 12–13 years. Regarding stroke, the ORs for age at menarche ≤ 11, 14–15, and ≥ 16 years were significantly higher (7%, 24%, and 94% increase, respectively) compared to age at menarche 12–13 years. Dose–response meta-analysis and one-stage random-effect cubic spline models confirmed the J-shaped risk pattern. Meta-regression indicated that age and BMI were not significant sources of heterogeneity. Sensitivity analyses and the absence of publication bias further supported the robustness of the findings. This study concludes that age at menarche is independently associated with CV events, with a J-shaped pattern. The findings underscore the significance of considering menarche age as an independent risk factor for CV events. Further research is warranted to validate these findings and explore potential underlying mechanisms.

## Introduction

Cardiovascular (CV) events are the leading cause of mortality and major morbidity in both developed and developing countries^1^. The age-standardized prevalence of CV events in men is higher than in women, however, it is responsible for causing approximately 35% of annual female mortality and is one of the most common reasons for disability-adjusted life-years lost among women^2,3^.

Despite its importance, CV events in women are inadequately acknowledged, and their risk factors remain understudied^4^. However, along with well-known cardiovascular risk factors such as smoking and obesity, there are other female-specific risk factors, such as reproductive age characteristics, which could also influence women’s CV disease risk throughout their lifespan^5^.

Age at menarche, defined as the age at first menstruation in adolescent girl, is one of the most important reproductive characteristics of women and is a milestone of pubertal development^6^. Emerging evidence suggests that the timing of menarche is associated with a higher risk of some cancers^7,8^, chronic disorders^9,10^, cardiometabolic disturbances^11–14^, higher adult body mass index (BMI)^15,16^ and hypertension^17^.

In addition, some studies specifically investigate the association between age at menarche and CV events. While certain systematic review and meta-analysis studies have indicated that an early onset of menarche is linked to an increased risk of all-cause mortality, the evidence concerning its association with mortality related to CV events or other CV events remains somewhat inconclusive^18,19^. Besides, in a separate systematic review study, Luijken et al. reviewed the data on the association between age at menarche and different subtypes of CVD^20^. They observed that among eight studies involving Caucasian populations, a consistent inverse linear relationship was reported between age at menarche (AAM) and cardiovascular disease (CVD) risk. However, a significant U-shaped relationship was observed in a large-scale study (n = 1,200,000)^21^. However, data from Asian populations yielded inconclusive results regarding the association between AAM and CVD risk. It should be noted that using different criteria for early age at menarche may lead to discrepancies between studies. In a pooled Analysis of Individual Patient Data of 307,855 women, Mishra et al.^22^ reported the U-shaped association between age at menarche and CVD. However, in a separate systematic review, Luijken et al.^20^ highlighted heterogeneity among the findings of available studies.

These findings suggest that the association between age at menarche and CV events is not entirely clear and needs to be precisely estimated. Therefore, the aim of this systematic review and meta-analysis of observational study was to assess the associations between age at the menarche and CV events among women.

## Material and methods

This review followed the Preferred Reporting Items for Systematic Reviews and Meta-analyses (PRISMA) reporting guidelines^23^. The trial protocol was registered in the International Prospective Register of Systematic Reviews (PROSPERO) under the registration number CRD42023453056.

The review question was framed using the PICO (population, intervention/index, control, and outcomes) statement as follows: P: all women who experience menarche which was classified into different monarchial age groups; I: age at menarche; C: women who experienced menarche at the normal age^24–26^; O: cardiovascular events including stroke, coronary heart disease and CV mortality.

### Eligibility criteria

This review considered all types of analytic observational studies and assessed the association between age at menarche and any cardiovascular events. Eligible studies were required to clearly define the age at menarche. Additionally, eligible studies needed to report an accurate number of CV events. To ensure the applicability of findings to the general population, studies focusing on women with severe diseases or serious conditions were excluded. Also, gray literature and non-original studies including reviews, commentaries, editorials, letters, meeting abstracts, case reports, conference proceedings, governmental or organizational reports, dissertations, theses, unpublished data, and presentations that did not provide accurate and clear data on research variables, were excluded. Articles not published in English were also excluded. Data for eligible articles in the press were requested from the study authors.

### Search strategy

A systematic computerized literature search of four electronic databases including PubMed, EMBASE, Scopus and Web of Science, covering the period from January 1, 2000, to October 31, 2023. A set of relevant terms was combined and used to narrow the search. Truncations were applied where appropriate, following the syntax rules of each database. Two filters, selecting only human studies and English publication, were applied. Additionally, a manual search in the references lists of selected studies and other relevant reviews was performed. The specific search strategy is presented in Supplementary Table 1, using PubMed as an example.

### Study selection and extraction

EndNote software (version X8, Clarivate Analytics, 2017 Boston, MA) was used to export identified references. After removing duplicates, titles, abstracts, and full texts were screened based on the aforementioned selection criteria. Two researchers (SB-G and RBY) completed all stages of the screening process independently, and discrepancies were resolved through discussion. If necessary, additional reviewers were consulted for further input. Throughout the review of abstracts and full-text articles, a list of references that did not meet the eligibility criteria was maintained, along with notes on the reasons for exclusions. Data on study characteristics, participant descriptions, association details, outcomes, and statistics were independently extracted by two reviewers. For missing relevant data, authors of eligible studies were contacted via email. The characteristics of included studies were summarized in Table 1.

**Table 1:** Characteristics of study population.

Authors, year of publication	Country	Study design	Sample size	Age at menarche	Age of assessment	Time period or follow-up time, if applicable	Actual age span covered in each study	Endpoint	Adjustment for analysis
Alonso de Leciñana et al. (2007)	Spain	Case–control study	Case: 430, Control: 905	< 12, 12, 13, ≥ 14	NM	NA	46 to 93 years	Stroke	Age-matched, adjusted for hypertension diabetes, hyperlipidemia, smoking, obesity
Bertuccio et al. (2007)	Italy	Case–control study	Case: 609, Control: 1106	< 12, 12, 13, 14, ≥ 15	NM	NA	18–79 years	Coronary heart disease	Age, study, education, body mass index, parity, menopausal status, age at menopause, smoking, coffee, alcohol, cholesterol, history of diabetes, obesity, hyperlipidemia, hypertension, use of HRT and family history of acute myocardial infarction in first degree relatives
Canoy et al. (2015)	UK	Prospective study	1,217,840	< 10, 11, 12, 13, 14, 15, 16, ≥ 17	Mean age of 56 (5) years	Mean of 11.6 years	NM	Stroke, coronary heart disease,	For year of birth, body mass index, height, smoking (never, past, and current smokers with consumptions of < 5, 5–9, 10–14, 15–19, 20–24, and ≥ 25 cigarettes per day), weekly alcohol consumption (0, 1–6, 7–14, and ≥ 15 units), frequency of strenuous exercise (rarely/never, once a week or less, and more than once a week), and socioeconomic status (fifths of Townsend index of deprivation)
Chang et al. (2011)	South Korea	Prospective study	3257	10–16, 17, 18, ≥ 19	≥ 55 y	1985 to 2005	NM	Cardiovascular mortality	Age at entry, body mass index, hypertension, drinking, smoking, education, and occupation
Chen et al. (2022)	China	Prospective study	6198	≤ 13, 14, 15, 16, ≥ 17	Mean age of 63.6 (9.9) years	5 years	NM	Composite outcome of CVD events of CHD, stroke, chronic heart failure, death	Age at recruitment (continuous), BMI (continuous), waist circumference (continuous), ethnicity (Han or other ethnicities), region (urban or rural), marital status (unmarried/widowed, married/cohabiting), education level (elementary or below, junior high school, high school or above), alcohol drinking (yes or no), smoking (yes or no), pharmacological treatment (yes or no), comorbidity (yes or no), family history of CVD (yes or no), ever pregnant (yes or no), contraceptive use (yes or no), breastfeeding experience (yes or no), and parity (0–1, 2, ≥ 3)
Cui et al. (2006)	Japan	Prospective study	37,965	≤ 13, 14, 15, 16, ≥ 17	40–79 years	10-year	40–79	Cardiovascular mortality	Age, smoking status (never, ex-, current 1–19, and ≥ 20 cigarettes/day), alcohol intake categories (never, ex-, current ethanol 1–22, 23–45, 46–68, and ≥ 69 g/day), marital status (married, widowed, divorced, single), type of menopause (natural, surgical, or unknown), education (primary school, junior high school, high school, college or more), histories of hypertension (no, yes) and diabetes (no, yes)
Day et al. (2015)	UK	Case–control Study	NM	8–11, 15–19	NA	NA	40–69 years	Angina, heart attack/myocardial, infarction, deep venous thrombosis	Principle components for socioeconomic position and adiposity/body composition
Hu et al. (2021)	China	Prospective study	16,504	≤ 12, 13, 14, 15, 16,17, ≥ 18		1976 to 1988, median of 12.0 years	NM	CV mortality	Age, diabetes, hypertension, dyslipidaemia, smoking, alcohol consumption, physical activity, body mass index, self-rated, health, education, job, family income, number of children and oral contraceptive pill use
Jacobsen et al. (2009)	USA	Prospective study	19,462	< 11, 11, 12, 13, 14, 15, 16, ≥ 17	55.1 years	11.1 years, range: 0–12 years	26–101 years	CV mortality	Age at enrolment
Jeong et al. (2023)	Republic of Korea	Prospective study	1,088,992	≤ 12, 13, 14, 15, 16, ≥ 17	Mean age: 43.8 ± 5.3 years (98.9%, < 55 years),	Mean follow-up of 8.3 years, 9,032,685.9 person-years	98.9%: < 55 years),	Myocardial infarction and ischaemic stroke	Age, plus additional adjustment for cardiovascular risk factors (income, smoking, alcohol consumption, regular exercise, body mass index, systolic blood pressure, total cholesterol, fasting glucose, hypertension, diabetes mellitus, and dyslipidemia) and plus additional adjustment for reproductive factors (age at menarche, duration of oral contraceptives use, duration of breast feeding, and parity)
Jeong et al. (2023)	Republic of Korea	Prospective study	1,224,547	≤ 12, 13–14, 15, 16, ≥ 17 years	60.8 ± 8.0	Median follow-up of 8.4 years	NM	Myocardial infarction and ischemic stroke	Cardiovascular risk factors (income, smoking, alcohol consumption, regular exercise, body mass index, systolic blood pressure, total cholesterol, fasting glucose, hypertension, diabetes mellitus, and dyslipidemia) and reproductive factors (parity, duration of breast feeding, duration of hormone replacement therapy, and duration of oral contraceptive use);
Jung et al. (2016)	Republic of Korea	Prospective study	66,104	≤ 12, 13–14, 15–16, ≥ 17		1996–2004 mean follow-up period of 12.4 years	NM	Atherosclerotic cardiovascular disease, stroke, and ischemic heart disease	Age, age squared, smoking status, body mass index, systolic blood pressure, total cholesterol, high-density lipoprotein cholesterol, exercise, socioeconomic status (premium), and diabetes
Kim et al. (2016)	Republic of Korea	Case–control study	Case: 178, Control: 509	< 14, 14–15, 16–17, ≥ 18	59.9 (11.4) years	NA	Aged 20 years and over	Obstructive Coronary Artery Disease	Age, diabetes mellitus, hypertension, dyslipidemia, estimated glomerular filtration rate, high-density lipoprotein cholesterol
Lakshman et al. (2009)	UK	Prospective study	15,807	8–11, 12, 13, 14, 15–18	Aged 40–79 yr	1993–1997 to 2007 median follow-up 10.6–12 yr	40–79 years	Coronary heart disease, stroke, other, CV events mortality	Age, physical activity, smoking, alcohol, educational level, occupational social class, oral contraceptive use, hormone replacement therapy, parity, body mass index, and waist circumference
Lee et al. (2019)	USA	Prospective study	648	≤ 10, 11, 12, 13, 14, ≥ 15	57.9 (12) years	Median of 6 years	NM	CV mortality	CVD risk factors of age, body mass index, diabetes mellitus, dyslipidemia, hypertension, history of smoking, and log serum amyloidand lifetime total estrogen exposure
Ley et al. (2017)	USA	Prospective study	73,814	≤ 10, 11, 12, 13, 14, 15, ≥ 16	NM	1980–2012 (1,467,987 person-years of follow-up)	30–55 years	Coronary heart disease or stroke	Alcohol intake, demographic, reproductive and medical history, lifestyle factors including ethnicity (European descent, Asian, Hispanic, and black), hormone therapy use (never, current, or past), oral contraceptive use (never, ever), parity (nulliparous, 1–2, or ≥ 3), family history of MI (excluded in stroke outcome analysis), family history of stroke (excluded in CHD outcome analysis), smoking (never, past, or current 1–14, 15–24, or ≥ 25 cigarettes/day), moderate-to-vigorous exercise (0, 0.01–1.0, 1.1–3.4, 3.5–5.9, or ≥ 6 h/week), Alternate Healthy Eating Index score (< 45, 45 to < 60, or ≥ 60), aspirin use (yes, no), alcohol intake (0, 0.1–4.9, 5.0–14.9, or ≥ 15.0 g/day), BMI (< 18.5, 18.5– 22.4, 22.5–24.9, 25.0–27.4, 27.5–29.9, 30.0–34.9, or ≥ 35.0 kg/m2), BMI at age 18 years (< 18.5, 18.5–22.4, 22.5–24.9, 25.0–27.4, 27.5–29.9, 30.0–34.9, or ≥ 35.0 kg/ m^2^), and menopause type (natural, surgical), history of diabetes mellitus, hypertension, and hypercholesterolemia
Liang al. (2021)	USA	Prospective study	264,546	< 12, 12, 13, 14, 15, ≥ 16	39–71 years	2006–2010	39–71 years	Composite outcome of CV events including heart attack, angina, or stroke	Age, race/ethnicity, Townsend deprivation index, smoking status, alcohol status, physical activity, menopause status, parity, BMI, healthy diet score, birth weight, assessment center, and history of cancer, CVD, diabetes, hypertension, and high cholesterol
Liu et al. (2018)	China	Cross-sectional study	7119	≤ 11, 12–13, 14–15, 16–17, ≥ 18	Mean age of 44.7 years	NA	14.3–106.9 years	Coronary heart disease, stroke	Age at enrollment (model 1), then adjustment for education level, marital status, employment status, ever active smoking, ever alcohol consumption, physical exercise, STPD, menopause, ever HT, ever OC use, parity, living children, ever breastfeeding, reproductive years, and pulse
Lozano-Esparza, et al. (2021)	Mexico	Prospective study	113,540	< 11, 11, 12, 13, 14, ≤ 15	Mean follow-up time of 9.2 years	2006–2017	Year of birth, ≤ 1955 to ≥ 1976	CV mortality	Year of birth and childhood factors including year of birth, indigenous ethnicity, parental occupation, meat consumption during childhood, number of older siblings, birthweight, body silhouette before menarche, and physical activity during adolescence
Lundblad et al. (2018	Norway	Prospective study	12,409	< 12, 12, 13, 14, > 15	NM	Mean of 18.7 years	25–94 years	CV mortality	Age, body mass index, physical activity, level of education and smoking history
Mueller et al. (2012)	Singapore	Prospective study	34,022	≤ 12, 13–14 15–16, ≥ 17	45–74	993–1998 til 2009, 460,374 person-years of follow-up	45–74 years	Coronary heart disease, stroke	Age at enrollment (continuous), year of interview (1993–95 vs. 1996–98), dialect (Hokkien vs. Cantonese), educational level achieved (no formal schooling, primary school, secondary school or above), smoking status (never, former, current), physical activity (≥ 2 h/week moderate or any strenuous vs. lower levels of activity), alcohol use (none, monthly, weekly, daily), vegetable fruit soy dietary pattern (quintiles), total energy intake (kcal; continuous), oral contraceptive use (never vs. ever), parity (0, 1–2, 3–4, ≥ 5 live births), menopausal status/age (pre-menopausal, < 50 years, ≥ 50 years), and hormone therapy use (estrogen or progesterone; never vs. ever) and baseline BMI (< 18.5, 18.5–21.4 (referent), 21.5–24.4, 24.5–27.4, ≥ 27.5)
Murakami et al. (2016)	Japan	Prospective study	1412	≤ 13, 14, 15, ≥ 16	Mean age of 65.9 ± 10.2 years	Median follow-up of 12.8 years	aged ≥ 35	Stroke, cerebral infarction	Age, BMI, Smoking status, Alcohol intake, Parity, Hormone replacement therapy, type of Menopause, Hypertension, Diabetes mellitus, family history of Heart disease, Hypercholesterolemia,
Ota et al. (2023)	Japan	Prospective study	54,937	9–12, 13, 14, 15, 16, 17, 18–20	NM	916,858 person-year follow-up	40–79 years old	Cardiovascular disease including deaths due to cardiovascular events, stroke, and ischemic heart disease	Age, body mass index, history of hypertension, history of diabetes, alcohol intake, smoking status, walking time, sport participation, sleeping hours, number of birth, menopausal age, and education level
Sun et al. (2023)	China	Prospective study	105,707	≤ 13, 16–17, ≥ 18	Mean age 55.31 ± 13.63 years	NM	January 1, 2016, to December 31, 2020	Valvular heart disease	Age, smoking status, systolic blood pressure, antihypertensive agents, diabetes mellitus, body mass index, high density lipoprotein cholesterol, and total cholesterol
Wu et al. (2014)	China	Prospective study	31,955	< 14, 14,15,16, ≥ 17		Median Follow-Up of 11.2 Years	40–70 years	CV mortality	Age at study enrollment (years), education (4 categories), occupation (4 categories), income (4 categories), marital status (yes/no), BMI (kg/m2), WHR (continuous), regular exercise (met/hour/year), current smoking (yes/no), current alcohol consumption (yes/no), number of live births, and age at menopause
Yang et al. (2017)	China	Prospective study	302,632	≤ 12, 13, 14, 15, 16,17, ≥ 18	Mean age of 50.5 years	7 years follow-up	30–79 years	Coronary heart disease, stroke, CV mortality	Age, systolic blood pressure, household income, smoking, alcohol drinking, BMI, physical activities (metabolic equivalent tasks), selfreported or screen-detected diabetes, and leg length as a marker of pre-pubertal growth, menopause status, parity, age at first birth, breastfeeding duration and OC use, smoking, alcohol consumption, diabetes at baseline and OCs usage
Zhang et al. (2019)	USA	Prospective study	75,359	≤ 11, 12–13, ≥ 14	NM	Median of 13 years	50–78 years	CV mortality	Age, race/ethnicities, bmi, smoking status, alcohol consumption, physical activity, education levels, hormone replacement therapy use, live births, cvd history, reasons for menstrual period stopped, and intervention arms
Zheng et al. (2016)	China	Cross-sectional study	13,242	≤ 12, 13, 14, 15, 16, ≥ 17	24–79 years	2009–2013	24–79 years	Brain stroke CHD, heart failure	Age, BMI and height
Zhu et al. (2023)	Netherlands	Prospective study	229,026	< 12, 12–13, > 13	Mean age: 56.5 years	11.8 (IQR: 11.1–12.6) years	37 to 73 years	Heart failure	Traditional cardiovascular risk factors, including age, ethnicity, Townsend index, body mass index, waist, alcohol consumption, smoking status, systolic blood pressure, blood pressure–lowering medication, total and low-density lipoprotein cholesterol, lipid-lowering medication, history of diabetes, history of CVD, use of hormone replacement therapy, use of oral contraceptives, and history of hysterectomy and/or oophorectomy

*NM* not mentioned, *NA* not applicable, *CV* cardiovascular.

### Exposure and outcomes of study

Age at menarche was defined as the age in whole years at the first menstrual period. This variable was initially categorized into four groups (age at menarche: ≤ 11, 12–13, 14–15, and ≥ 16 years, with the reference group being women who were aged 12–13 years at menarche). The outcome of study included the specific subtypes of CV events including coronary heart disease, stroke and CV mortality. The criteria for stroke included subarachnoid hemorrhage, ischemic stroke, intracerebral hemorrhage, and unspecified stroke. Coronary heart disease was defined as myocardial infarction, coronary artery bypass graft surgery, or percutaneous coronary intervention and other coronary disease.

### Quality appraisal and statistical analysis

Two authors (CFM and SB-G) independently conducted a thorough critical appraisal of the selected studies. Any disagreements or discrepancies that arose during this process were resolved through discussion. In cases where necessary, other reviewers (IS and RB-Y) were consulted. The Newcastle–Ottawa Scale (NOS) was used for methodological structures and result presentation of the studies^27^. This scale includes three criteria: (i) participant selection (maximum of four stars); (ii) comparability of study groups (maximum of two stars) and (iii) assessment of outcome or exposure (maximum of three stars) for the outcome/exposure category. Studies with scores above 7 were considered high quality, those with scores between 4 and 7 were categorized as moderate quality, and those with scores less than 4 were judged as low quality. However, we planned to conduct a subgroup analysis by including and excluding results from studies of low quality, if any such studies were identified.

### Statistical analysis

All statistical analysis was performed using STATA software (version 14; STATA, INC., College Station, TX, USA). We conducted two types of analyses. First, to estimate pooled Odds Ratio (OR) and 95%CI of the outcomes of interest in menarche age groups versus controls, we employed DerSimonian & Laird and inverse variance methods to run random/fixed effect models. It is important to note that raw data were extracted from different types of studies such as case–control, prospective and cross-sectional. For each one, the OR (SE log OR) were estimated, separately. However, some approximation was considered for relative risks and Hazard ratio with odds ratios^28^. Heterogeneity was quantified using the I-squared measure and Chi-squared test. In this case, an I-squared value exceeding 50% was considered medium to high heterogeneity. A significant result in the Chi-squared test was also considered as heterogeneity. In the presence of significant heterogeneity, a random effect model was applied.

Forest plots were generated to display the included studies for estimation of pooled OR (95% CI). Publication bias was assessed via Begg’s and Harbord’s tests. Sensitivity analysis was run to investigate the influence of each individual study on the overall meta-analysis summary estimate. Additionally, meta-regression analysis was conducted to explore the potential sources of heterogeneity related to age and BMI. Significant level was set at 0.05.

Second, dose Response meta-analysis was also performed to consider menarche age as continues variable and show the trend of risks according to the exposure dose. dose One-stage random-effect (for both intercept and slope coefficients) dose–response Linear and restricted cubic spline with the three selected knots models were fitted to detect the trend of risks considering menarche age 13 as reference age^29^.

Linear dose response model consider age at menarche as a continuous variable so the exponential of regression coefficient shows the linear trend. On the contrary, the cubic spline model considers a non-linear association between age at menarche and risk of adverse events. Based on the model represented by Harrell FE Jr.^30^.

Restricted cubic spline models with three knots (at percentiles 10, 50 and 90) defines as,

$$y_{i} = \left( {\beta_{1} + b_{i1} } \right)\left( {x_{1} i - x_{1} i_{0} } \right) + \left( {\beta_{2} + b_{i2} } \right)\left( {x_{2} i - x_{2} i_{0} } \right) + \in_{i} .$$

We consider menarche age 13 year as referent, so exp (B_1_) and exp (B_2_) showed the odds ratios of adverse events at age lower 13 and upper 13 year, respectively.

## Results

### Identification of literature

**Figure 1:**
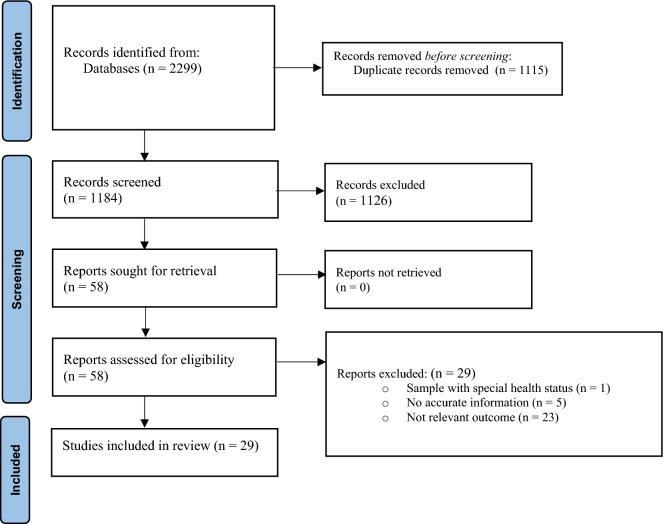
PRISMA flow diagram of the selection process.

Through electronic searches, we identified 2299 unique articles. No additional studies were identified through manual searches or contact with authors. We excluded duplicates that appeared in multiple databases. Subsequently, we evaluated the titles and abstracts of the remaining articles, resulting in 58 articles for further evaluation. After screening the full texts of these articles to ensure they meet the eligibility criteria, we included 29 articles^14,21, 31–57^ in the current review. The PRISMA diagram is presented in Fig. 1.

### Characteristics of included studies

The main characteristics of the included studies are presented in Table 1. Of the 29 included articles, data were derived from two independent case–control studies^31,32^, one cross-sectional study^37^ and the remaining studies were prospective. In total, these studies, involving a total of 4,931,160 participants, focused on adult women aged 18 years or older.

Age at menarche was assessed using self-report questionnaire in all studies. The length of recall of age at menarche was heterogeneous and predominantly reported in middle age. The included studies were published between 2006 and 2023, covering a study period of almost 50 years (1976–2023). A total of seven studies were conducted in Europe (including Spain^31^, Italy^32^, UK^14,21,46^, Norway^39^, Netherlands^53^), five in the USA^34–36,40, 47^, one in Mexico^38^) and 16 in Asia, including (China^33,37,42, 50–52,55^, South Korea^41,44,45,54,56^, Japan^43,49,57^, Singapore^48^).

The quality appraisal of the included studies has been presented in Supplementary Tables 2–4. Among them, a total of 23 studies were judged as high quality^14,21,33–36,38–44,46–51,53, 54,56,57^; six were rated as moderate quality^31,32,37,45,52,55^; and none were considered low quality. As no study was assessed as low quality, all studies included in the final analysis. The results of Begg’s and Harbord’s tests suggested no publication bias, however lower number of studies should be considered (Table 2).

**Table 2:** Results of heterogeneity, publication bias estimation, and pooled odds ratio (95% CI) of cardiovascular events based on various study population and specific CV events.

Age at menarche groups^€^															
Outcome	≤ 11 years					14–15 years					≥ 16 years				
	Heterogeneity		**Publication bias tests		OR (95% CI)	Heterogeneity		Publication bias tests		OR (95% CI)	Heterogeneity		Publication bias tests		OR (95% CI)
	I-squared	*Chi- squared	Begg	Harbor		I-squared	Chi- squared	Begg	Harbo		I-squared	Chi- squared	Begg	Harbor	
CV mortality	81.5%	27.00, d.f. = 5, p = 0.000	0.94 (0.348)	0.35 (0.743)	1.03 (0.85–1.24)	76.9%	17.34, d.f. = 4, p = 0.002	− 0.98 (0.327)	− 1.75 (0.179)	**1.37 (1.14–1.64)**	87.0%	30.74, d.f. = 4, p = 0.000	− 0.98 (0.327)	− 0.04 (0.972)	**1.64 (1.20–2.24)**
Coronary heart disease	96.0%	100.60, d.f. = 4, p = 0.000	0.00 (1.00)	-1.63 (0.201)	.98 (0.74–1.29)	94.6%	92.68, d.f. = 5, p = 0.000	− 1.24 (0.216)	− 1.67 (0.146)	1.05 (0.87–1.28)	98.3%	177.08, d.f. = 3, p = 0.000	− 1.35 (0.176)	− 1.54 (0.184)	**1.35 (1.03, 1.76)**
Stroke	22.7%	3.88, d.f. = 3, p = 0.275	0.00 (1.00)	-0.9 (0.429)	^**@**^**1.07 (1.04–1.11)**	81.1%	15.86, d.f. = 3, p = 0.002	− 1.32 (0.188)	− 0.82 (0.460)	**1.24 (1.10–1.41)**	96.9%	98.13, d.f. = 3, p = 0.000	− 1.35 (0.176)	− 1.66 (0.159)	**1.94 (1.38, 2.71)**

^€^The reference group for age at menarche was set as 12–13 years.

*Chi- squared statistic, degree of freedom, P-value.

**Statistic (z and t score) (P-value) for Begg and Harbord tests, respectively.

^@^Obtained from fixed effect model.

Bold values in the results indicate statistical significance.

### Result of meta-analysis

For the meta-analysis, we included studies^21,31–35, 37–40,53,54,57^ in which the classification of age at menarche was compatible with the following categories: ≤ 11 years, 12–13 years (reference group), 14–15 years, and ≥ 16 years.

**Figure 2:**
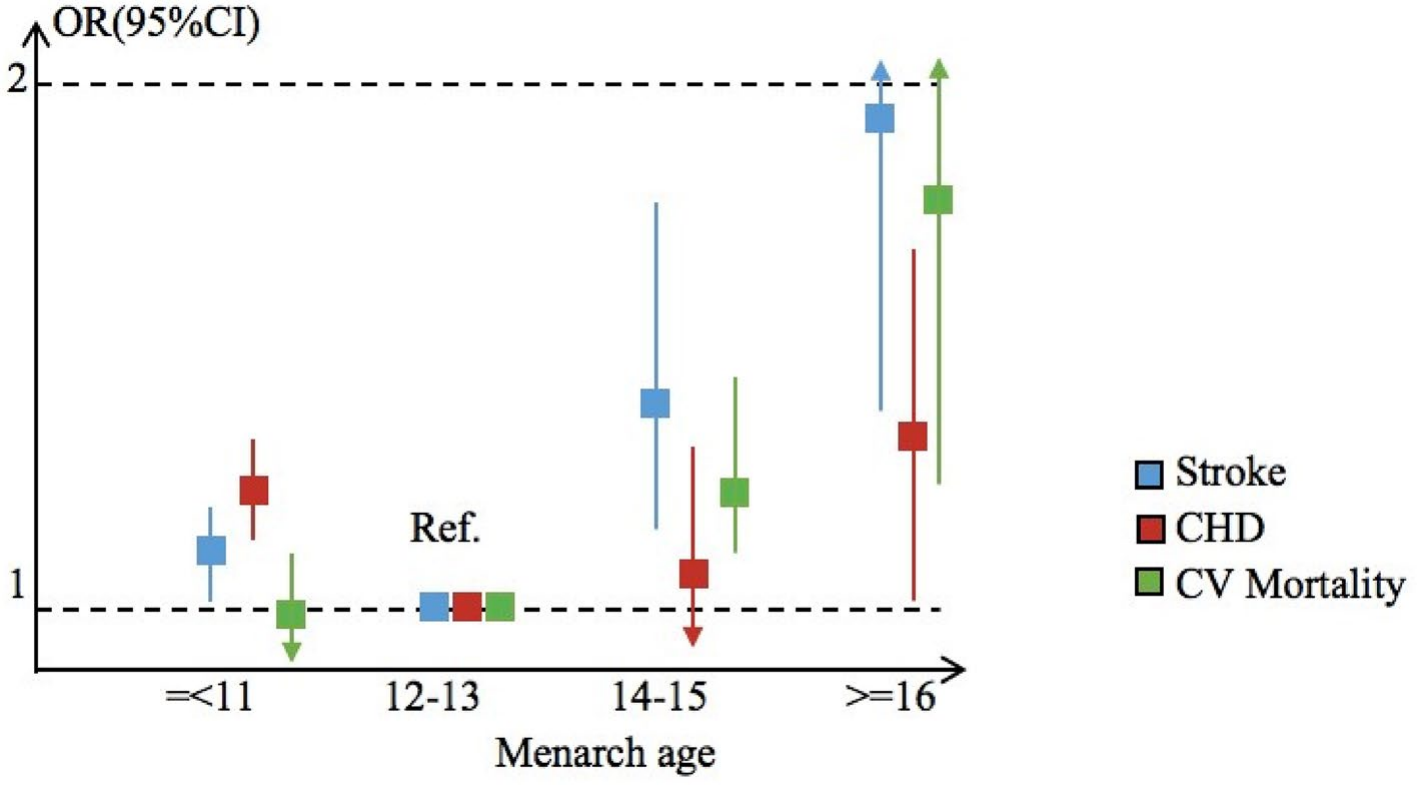
Pooled Odds Ratio (OR) and 95% confidence interval (CI) of cardiovascular (CV) events b age at menarche.

**Figure 3:**
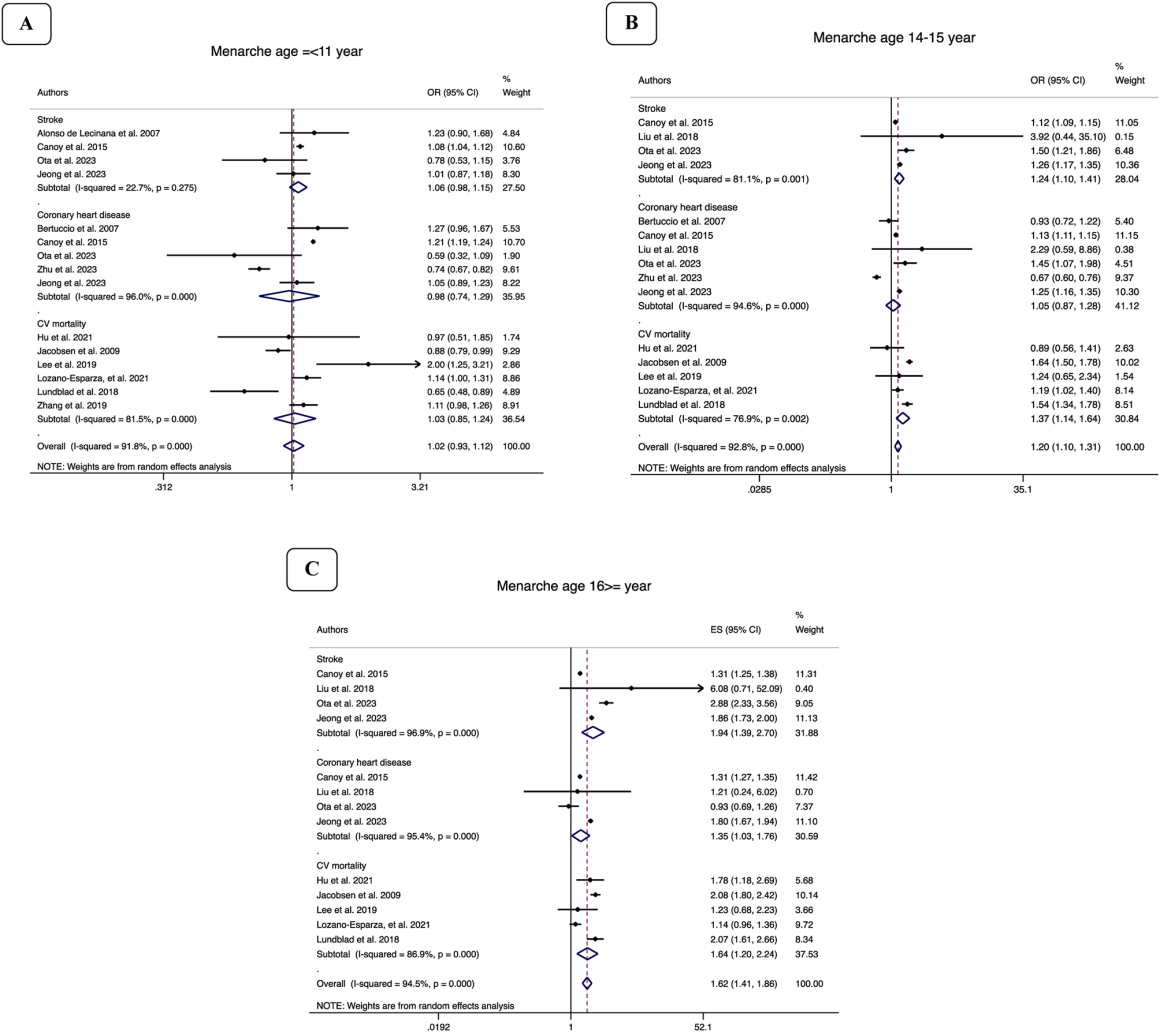
Forest plot of pooled OR and 95% confidence interval (CI) of cardiovascular events (**A**) among women with age at menarche ≤ 11 years compared to those with age at menarche 12–13 years (**B**) among women with age at menarche 14–15 years compared to those with age at menarche 12–13 years (**C**) among women with age at menarche ≥ 16 years compared to those with age at menarche 12–13 years.

Results of meta-analysis revealed a J-shaped association between age at menarche and the outcomes of CV events (Fig. 2), and women with an age at menarche of 12–13 years had a lower risk than other groups. Results are presented Table 2 and Fig. 3A–C.

The pooled odds of CV mortality in age at menarche categories 14–15 years and ≥ 16 years were 37% (OR: 1.37, 95% CI 1.14–1.64, I^2^: 76.9%) and 64% (OR: 1.64, 95% CI 1.20—2.24, I^2^: 87%) higher than referent age at menarche 12–13 years. Although it was higher, but no statistically significant difference was found in the risk of CV mortality between individuals with age at menarche ≤ 11 years and those with age at menarche 12–13 years.

As well, the pooled odds of coronary heart disease in age at menarche ≥ 16 years were significantly 35% (OR: 1.35, 95% CI 1.03—1.76, I^2^: 95.4%) higher than among those with age at menarche 12–13 years. No statistically significant difference was found in the risk of coronary heart disease between individuals with age at menarche ≤ 11 years or 14–15 years and those with age at menarche 12–13 years.

Regarding the stroke, the pooled odds of stroke in age at menarche ≤ 11, 14–15 and ≥ 16 years were significantly 7% (OR: 1.07, 95% CI 1.04–1.11, I^2^: 22.7%), 24% (OR: 1.24, 95% CI 1.10–1.41, I^2^: 81.1%), 94% (OR: 1.94, 95% CI 1.39–2.70, I^2^: 96.9%), higher than among those with age at menarche 12–13 years, respectively.

Secondary, using dose response meta-analysis, we included all studies^14,21,31–57^ used various classification of age at menarche. Results of dose response meta-analysis were also confirmed the obtained results. Considering AIC, one stage random effect Cubic spline model had better fit on data, showing J-shaped risk of CV events according to the menarche age considering 13 years as reference age (Supplementary Table 4 and Supplementary Fig. 3).

Results of restricted cubic spline estimated positive values of regression coefficients (B1 and B2) which interpreted that comparing age at menarche 13, lower and upper menarche age had an increasing trend of risk for a dose of 1 unit (menarche age). Compare to menarche age lower 13, odds ratio of stroke, CHD and CV mortality increase by 5% (OR: 1.05, 95% CI 0.94–1.11), 5% (OR: 1.05, 95% CI 0.97–1.14), and 7% (OR: 1.07, 95% CI 0.95–1.20), respectively. Age at menarche upper 13 year also showed the same increasing trend of risk of stroke, CHD and CV mortality increase by 4% (OR: 1.04, 95% CI 0.99–1.15), 2% (OR: 1.02, 95% CI 0.94–1.12), and 4% (OR: 1.04, 95% CI 0.94–1.16), respectively. The p-value for testing non-linearity (H0: β2 = 0) was significant for stroke, however for the rest of outcomes results showed lower power (Table 5-supplementary).

Results of meta-regression also showed that age and BMI at the time of recruitment of the study were not significant sources of heterogeneity (Supplementary Fig. 1). Results of sensitivity analyses showed that no single study essentially changed the pooled odds ratio of all outcomes (Supplementary Fig. 2). No publication bias was also detected (Table 2).

## Discussion

We have provided quantitative estimates for the associations between age at menarche and cardiovascular events after adjusting for confounding factors of age and BMI through a systematic search and comprehensive meta-analysis. The results of meta-analysis revealed a J-shaped association between age at menarche and the outcomes of CV events. Women with an age at menarche of 12–13 years had a lower risk compared to other groups with younger (≤ 11 years) or older (14–15 years and ≥ 16 years) age at menarche. However, individuals with age at menarche of 16 years and older exhibited the highest risk of cardiovascular events. Subgroup analysis revealed similar J-shaped associations for specific CV events including stroke, coronary heart disease and CV mortality. Notably, the magnitude of the risks for CV mortality was stronger than that observed for coronary heart disease and stroke. This finding further supports that age at menarche may be an independent risk factor for CV events later in life.

Cardiovascular events continue to be a significant causes of mortality and morbidity among women^58^. In addition to some traditional risk factors such as diabetes mellitus, smoking and obesity, a number of clinical conditions exclusive to women have been demonstrated to elevate the risks of CV events. In this respect, there is evidence showing the association between women’s reproductive age including age at menopause and menarche and the subsequent risk of CV events^22,47,59,60^. Menopause marks the end of the reproductive period and is recognized as one of the important CVD risk factors among women^61,62^. Menarche, on the other hand, is a marker of puberty signifies the onset of ovarian and other endocrine functions relating to reproduction. Nevertheless, the results of studies focusing on the association between CV events and age at menarche were controversial, and the current meta-analysis study contributes to the clarification of conflicting results reported by previous studies.

Our findings suggest that both an age at menarche of less than 12 years and an age at menarche of later than 13 years may contribute to an increased risk of CV events. This trend appears to be more pronounced in individuals with an age at menarche of 16 years and older. Our findings are consistent with earlier studies that demonstrated the association between age at menarche and CV events. In agreement with this study^22^, Mishra et al. in a pooled analysis of individual patient data from 12 Studies, showed that short reproductive life span (< 33 years) was associated with an increased risk of CVD events in midlife. Women who had both a short reproductive life span and early menarche (age ≤ 11 years) had the most pronounced risk of CVD events. On the other hand, using the similar criteria for early and late menarche definition as in our study, they reported the U- shaped association between age at menarche and CVD, with a higher risk of CVD for both early menarche (age ≤ 11 years) and late menarche (age ≥ 15 years).

In a separate systematic review study, Luijken et al. (2017) reviewed the data on the association between age at menarche and different subtypes of CVD^20^. They noted that among eight studies involving Caucasian populations, an inverse linear relationship was consistently reported between age at menarche (AAM) and cardiovascular disease (CVD) risk. However, a significant *U*-shaped relationship was observed in a large-scale study (n = 1,200,000)^21^. However, data from Asian populations were characterized by inconclusive results regarding the association between AAM and CVD risk. It should be noted that using different criteria for early age at menarche may lead to discrepancy between studies. In another systematic review and meta-analysis study, Charalampopoulos et al. reported that while no significant association was observed between an earlier age at menarche and CV mortality (HR = 1.05 (95% CI 0.90, 1.21), however, each 1-year increase in age at menarche was associated with a 3% lower relative risk of total CV mortality^18^.

While the precise mechanisms underlying the association between earlier age at menarche and CV events in the future are not entirely elucidated, there are critically potentially mediating factors indicating that an earlier age at menarche is associated with increased risk of childhood and adulthood obesity, hypertension, and metabolic syndrome^15,63–66^. Furthermore, history of low birth weight, and rapid infancy growth^67–69^ are critical mediating factors for the relation of early menarche with risks of coronary and CV events. Besides, emerging evidence indicated that genetic components influencing both puberty timing and BMI could act as a shared genetic connection that might explain the relationship between the age at menarche and the risk of developing cardiovascular disease^70^. Recently, Ardissino M, et al.^60^, provided genetic evidence to support that earlier menarche are associated with higher risk of atrial fibrillation, coronary artery disease, heart failure, and stroke in women. Utilizing Mendelian randomization, the authors established a causal relationship between reproductive factors and cardiovascular diseases in the female population. They showed that earlier genetically predicted age at menarche increased risk of coronary artery disease (OR per year, 1.10, 95% CI 1.06–1.14) and heart failure (OR, 1.12, 95% CI 1.07–1.17); both associations were at least partly mediated by BMI.

The results of the study demonstrated that later age at menarche was associated with a higher risk of composite and subtypes of CV events. The higher risk of CV events in these women may be partly explained by shorter reproductive lifespan and subsequently shorter exposure to estrogen. In this respect, it is reported that estrogen, particularly estradiol (E2), acts as a mediator in CVD protection by promoting angiogenesis, vasodilation and decreasing reactive oxygen species, oxidative stress, and fibrosis^71,72^. In agreement with this finding, Mishra et al. in a systematic review and meta-analysis reported that a shorter reproductive lifespan was associated with a higher risk of CVD events, particularly stroke^73^. Consistent with our findings, they reported no evidence of an association between early age at menarche and CVD mortality (RR: 1.05, 95% CI 0.95, 1.14; heterogeneity I^2^ = 0.2%, p = 0.391). However, in contrast to our finding, they reported that early age at menarche was not significantly associated with a moderately higher risk of stroke events (RR: 1.17, 95% CI 0.20, 2.14; heterogeneity I^2^ = 69.6%, p = 0.070) (5, 28). We, on the other hand, found that the risk of stroke in individuals with age at menarche ≤ 11 years was significantly 8% higher (OR: 1.08, 95% CI 1.04–1.12, I^2^ = 0) than among those with age at menarche between 12 and 13 years. This discrepancy may be related to the fact that the definition of early age at menarche in these two meta-analyses differs. In our meta-analysis, we used the precise definition, where age at menarche was defined as being less than or equal to 11 years. In the Mishra study, they used the definitions of each individual study, including two studies that used age at menarche ≤ 13^49^ and ≤ 12 years^44^, which may have affected the final findings.

In the current meta-analysis, we found no publication bias. Meta-regression showed that the age and BMI of participants were not the source of heterogenicity. However, the subgroup analysis based on subtypes of CV events revealed decreased I^2^ among them, suggesting that the type of CV events contributed to those heterogeneities. Despite this, concerns remain regarding the lower power of meta-regression analysis and sample size.

In addition, ethnicity appears to play a significant role in determining the age of menarche. Generally, some studies have indicated that the natural mean age at menarche is higher in Asian populations when compared to Caucasian populations^20^. Furthermore,—African-American girls tend to experience menarche earlier than their Caucasian counterparts^74,75^. Nevertheless, our search did not yield relevant studies concerning the relationship between age at menarche and cardiovascular (CV) events within African and Middle-East Asian populations, which may exhibit different characteristics of the Western countries or East Asian populations. Hence, due to a lack of data, we could not perform such a sub-analysis based on ethnicity.

Our results may have potential public health implications. In this respect, data on the onset of menstruation as a potential risk factor for CVD may be valuable for intervention strategies targeting modifiable factors, aiming at improved CV health outcomes in the long-term.

Our study had some limitations. Despite conducting an extensive literature search, certain unpublished studies that, those written in languages other than English, or those presenting age at menarche as a continuous variable were not included. All of the original studies included relied on self-reported data for age at menarche, potentially introducing results with recall biases. Furthermore, the length of recall of age at menarche was heterogeneous.

However, to reduce recall bias, various strategies were implemented by separate included studies. These strategies included assessing the correlation with age at puberty, calculating the duration of reproductive years by subtracting the age at menarche from the age at menopause, restricting the analysis to women who consistently provided reports without hesitation or had documented gynecologic histories, and evaluating the accuracy of self-reported age at menarche. These approaches enhanced the validity and reliability of self-reported age at menarche. As such, numerous studies have indicated that self-reported age at menarche is sufficiently accurate for utilization in epidemiological investigations studies^76,77^. All the studies included had an observational framework, which implies that residual or unmeasured confounding might not have been entirely controlled. Despite the stringent inclusion criteria that led to the inclusion of a limited number of studies in this meta-analysis, the findings of our meta-analysis could give a better, more unbiased and professional impression and offering up-to-date evidence on this crucial topic. Moreover, it's important to highlight that the majority of included studies in the current meta-analysis were conducted with a population-based approach, as a representative of general population characteristics with minimizing the potential for selection bias. As a result, the outcomes of this study are applicable for general population extrapolation. It is worth mentioning that, for most studies with different study design we extracted the raw data and estimated the OR directly. However, there were some concerns regarding the overestimation or of pooled estimates of OR when it comes to cohort studies. Problems may arise, if the odds ratio is misinterpreted as a risk ratio or hazard ratio in cohort studies. For exposures that increase the chances of events, the odds ratio will be larger than the risk ratio, so the misinterpretation will tend to overestimate the exposure effect, especially when events are common (risks of events more than10%) For exposure that reduce the chances of events, the odds ratio will be smaller than the risk ratio, so that, again, misinterpretation overestimates the effect of the exposure^78,79^. There were 3 cohort studies with prevalence over 10% regarding the CV mortality outcome, although results of sensitivity analysis did not show strong influence of these studies. In this study, we have also explored a J-shape pattern for the risk of cardiovascular events by menarche age using a restricted random-effect cubic spline model with three knots, although based on the figures upper limit of 95% CI for cubic spline model showed the J-shaped pattern much better. This inconsistency might be a matter of sparse data especially in lower age 13 (stroke: 18 records lower 13 year out of 65 upper 13 year, CHD: 23 records out of 78 and CV mortality: 17 records out of 54), which model could not fit well and detect true pattern due to insufficient evidence. The p-value for testing non-linearity (H0: β2 = 0) was not significant for the outcomes except stroke, it might be due to the low power. In addition, Stone and Koo proved cubic spline functions have a drawback that behaved poorly in the tails, that is before the first knot and after the last knot. They cite advantages of constraining the function to be linear in the tails, called natural splines^80^.

## Conclusion

In conclusion, we observed a J-shaped association between age at menarche and CV events. The risk was lowest for menarche at 12–13 years of age, increasing with younger (≤ 11 years) and older (14–15 years and ≥ 16 years) age at menarche. Individuals with age at menarche of 16 years and older exhibited the highest risk of CV events. This finding further supports that age at menarche may be an independent risk factor for CV events later in life. Future studies are warranted to confirm these findings and to explore the potential underlying mechanism linking CV events and onset of age at menarche.

### Supplementary Information

Supplementary Information.

## Data Availability

All data generated or analyzed during the present study are included in this published article.
