# Supplementary material for: The J shaped association of age at menarche and cardiovascular events: systematic review and meta-analysis
Source: Sci Rep. 2024 Feb 1;14:2695. doi: 10.1038/s41598-024-53011-5 (PMC10834967; doi:10.1038/s41598-024-53011-5)
Supplement: Supplementary file 1 — Supplementary Information. [file 41598_2024_53011_MOESM1_ESM.docx]

**Supplementary material**

Table 1: The specific search strategy will be (taking PubMed as an example):

| Search query | Search keywords (title and abstract) |
| --- | --- |
| 1 | ("age at menarche"[Title/Abstract] OR "menarche"[Title/Abstract] OR "menarcheal age"[Title/Abstract] OR "menstruation"[Title/Abstract] OR "menstrual"[Title/Abstract] OR "puberty"[Title/Abstract] OR "Early menarche"[Title/Abstract] OR "precocious puberty"[Title/Abstract] OR "Late menarche"[Title/Abstract] OR "delayed puberty"[Title/Abstract] OR "late puberty"[Title/Abstract] OR "early puberty"[Title/Abstract]) OR "reproductive"[Title/Abstract]) OR "Reproduction"[Title/Abstract]) AND ((humans[Filter]) AND (english[Filter]))  Sort by: Most Recent |
| 2 | ("cardiovascular"[Title/Abstract] OR "cardiovascular disease"[Title/Abstract] OR "cardiovascular events"[Title/Abstract] OR "myocardial infarction"[Title/Abstract] OR "MI"[Title/Abstract] OR "Coronary heart disease"[Title/Abstract] OR "IHD"[Title/Abstract] OR "Ischemic heart disease"[Title/Abstract] OR "angina"[Title/Abstract] OR "heart attack"[Title/Abstract] OR "coronary arterial disease"[Title/Abstract] OR "stroke"[Title/Abstract] OR "Cerebral Infarction"[Title/Abstract] OR "hemorrhagic stroke"[Title/Abstract] OR "ischemic stroke"[Title/Abstract] OR "CVD"[Title/Abstract] OR "Cerebrovascular disease"[Title/Abstract] OR "Heart failure"[Title/Abstract] OR "Heart valve disease"[Title/Abstract] OR "Aortic stenosis"[Title/Abstract] OR "Mitral regurgitation"[Title/Abstract] OR "cardiovascular death"[Title/Abstract] OR "cardiovascular mortality"[Title/Abstract] OR "cardiovascular morbidity"[Title/Abstract] OR "Pulmonary hypertension"[Title/Abstract] OR "HTN"[Title/Abstract] OR "hypertension"[Title/Abstract]) AND ((humans[Filter]) AND (english[Filter]))  Sort by: Most Recent |
| Final search query | (1) AND (2)  Filters: Humans, English, from 2000 - 2023  Sort by: Most Recent |

Supplementary table 2. Quality assessment of included studies using the Newcastle–Ottawa Quality Assessment Scale for prospective studies.

|  | SELECTION | | | | COMPARABILITY | OUTCOME | | | Total scores |
| --- | --- | --- | --- | --- | --- | --- | --- | --- | --- |
| Author, year, code | Representativeness of the exposed cohort  *( truly representative* or somewhat representative in the community**) | Selection of the non-exposed cohort  ( *drawn from the same community as the exposed cohort**) | Ascertainment of exposure  *(secure record (eg surgical records)* or structured interview)** | Demonstration that the outcome of interest was not present at the start of study * | Comparability of cohorts on the basis of the design or analysis  *(A: Study controls for age and/or Sex* and*  *B: Study controls for other confounders*)* | Assessment of outcome  *(A: Independent blind *assessment*  *B: Record linkage*)* | long enough for outcomes to occur* | Adequacy of follow up of cohorts  *(complete follow up - all subjects accounted for * OR lost to follow up unlikely to introduce bias** |  |
| Canoy et al., 2014 | * | * | * | * | ** | * | * | * | 9 |
| Chang et al., 2011 | * | * | * | * | ** | * | * | * | 9 |
| Chen et al., 2022 | * | * | * | * | ** | * | * | * | 9 |
| Cui et al. 2006 | * | * | * | * | ** | * | * | * | 9 |
| Day et al., 2015 | * | * | * | * | ** | * | * | * | 9 |
| Hu et al., 2021 | * | * | * | * | ** | * | * | * | 9 |
| Jacobsen et al., 2009 | * | * |  | * | ** | * | * | * | 8 |
| Jung et al., 2015 | * | * | * | * | ** | * | * | * | 9 |
| Kim et al., 2016 | * | * | * | * | ** | * | - | - | 7 |
| Lakshman et al., 2009 | * | * | * | * | ** | * | * | * | 9 |
| Lee et al., 2019 |  | * | * | * | ** | * | * | * | 8 |
| Ley et al., 2017 | * | * | * | * | ** | * | * | * | 9 |
| Liang et al, 2021 | * | * | * | * | ** | * | * | * | 9 |
| Lozano- Esparaza et al., 2021 | * | * | * | * | ** | * | * | * | 9 |
| Lundblad & Jacobsen, 2018 | * | * | * | * | ** | * | * | * | 9 |
| Mueller et al., 2012 | * | * | * | * | ** | * | * | * | 9 |
| Murakami et al., 2016 | * | * | * | * | ** | * | * | * | 9 |
| Wu et al., 2014 | * | * | * | * | ** | * | * | * | 9 |
| Yang et al., 2016 | * | * | * | * | ** | * | * | * | 9 |
| Zhang et al., 2019 | * | * | * | * | ** | * | * | * | 9 |
| Zheng et al., 2016 | * | * | * | * | ** | * | - | - | 7 |
| Ota et al, 2023 | * | * | * | * | ** | * | * | * | 9 |
| Jeong et al, 2023 | * | * | * | * | ** | * | * | * | 9 |
| Sun et al, 2023 | ? | * | * | * | ** | * | - | - | 7 |
| Zhu et al, 2023 | * | * | * | * | ** | * | * | * | 9 |
| Jeong et al, 2023 | * | * | * | * | ** | * | * | * | 9 |

Supplementary table 3. Quality assessment of the selected studies using the Newcastle–Ottawa Quality Assessment Scale for Case-Control studies

|  | SELECTION | | | | COMPARABILITY | Exposure | |  | Total scores |
| --- | --- | --- | --- | --- | --- | --- | --- | --- | --- |
| Author, year, code | Is the case definition adequate?  (*with independent validation**) | Representativeness of the cases (*consecutive or obviously representative series of cases**) | Selection of Controls (*community controls**) | Definition of Controls (*no history of disease, endpoint**) | Comparability of cases and controls on the basis of the design or analysis  *(A: study controls for Select the most important factor.*and B: study controls for any additional factor*)* | Ascertainment of exposure (*secure record. eg surgical records**) | Same method of ascertainment for cases and controls* | Non-Response rate (*same rate for both groups)** |  |
| Alonso de Lecinana, et al., 2007 | * |  |  | * | ** | * | * |  | 6* |
| Bertuccio et al., 2007 | * |  |  | * | ** | * | * |  | 6* |

Supplementary table 4. Quality assessment of included studies using the Newcastle–Ottawa Quality Assessment Scale for adapted for Cross-Sectional studies.

|  | SELECTION | | | | COMPARABILITY | Outcome | | Total scores |
| --- | --- | --- | --- | --- | --- | --- | --- | --- |
| Author, year, code | Representativeness of the samples*  (*consecutive or random sampling of cases* or Somewhat representative of the average* *with non-random sampling*)* | Sample size  (J*ustified and satisfactory*)* | Non-responders  *(The response rate is satisfactory (≥95%).)** | Ascertainment of the exposure/survillence tool  *(Validated screening/surveillance tool*) ** or  (Non-validated but the tool is available or described*) | The potential confounders were investigated by subgroup analysis or multivariable analysis  (A: study controls for age and/or BMI *and  B: control for any additional factor*) | Assessment of the outcome  a) Independent blind assessment**  b) Record linkage*  c) Self report* | Statistical test (clearly described and appropriate*) |  |
| Liu et al., 2018 | * | * |  | * | ** | * | * | 7 |

**Results of Dose response meta-analysis**

Supplementary table 5: Results of Dose response meta-analysis, One-stage random-effect dose-response Linear and Restricted Cubic spline models

| Models | | exp(B) | | 95%CI exp(B) | | Model AIC | ^@^p-value non-linearity test | ^@@^p-value heterogeneity  test |
| --- | --- | --- | --- | --- | --- | --- | --- | --- |
| Stroke | | | | | | | | |
| Model 1 | | 1.07 | | (1.03, 1.13) | | 136.27 | -- | <0.001 |
| Model 2 | | exp(B1) | exp(B2) | 95%CI exp(B1) | 95%CI exp(B2) | 39.63 | 0.027* | <0.001 |
|  |  | 1.05 | 1.04 | (.94, 1.11) | (.99, 1.15) |  |  |  |
|  | CHD | | | | | | | |
| Model 1 | | 1.06 | | (1.01, 1.12) | | 854.10 | -- | <0.001 |
| Model 2 | | exp(B1) | exp(B2) | 95%CI exp(B1) | 95%CI exp(B2) | 421.73 | 0.081 | <0.001 |
|  |  | 1.05 | 1.02 | (.97, 1.14) | (.94, 1.12) |  |  |  |
|  | CV Mortality | | | | | | | |
| Model 1 | | 1.08 | | (1.01, 1.07) | | 248.84 | -- | <0.001 |
| Model 2 | | exp(B1) | exp(B2) | 95%CI exp(B1) | 95%CI exp(B2) | 212.66 | 0.158 | <0.001 |
|  |  | 1.07 | 1.04 | (.95, 1.20) | (.94, 1.16) |  |  |  |

Model 1: One-stage random-effect dose-response linear model

Model 2: One-stage random-effect dose-response restricted cubic spline model

^@^ P-value for testing non-linearity (H_0_: β_2_=0)

^@@^Chi-squared test of heterogeneity

Supplementary figure 1: Bubble plots of meta-regression results

|  |  |
| --- | --- |
|  |  |

Supplementary figure 2: Sensitivity analysis by outcomes and menarche age =<11, 14-15 and >=16 years.

| Stroke: =<11 | 14-15 | >=16 |
| --- | --- | --- |
|  |  |  |
| CV Mortality: =<11 | 14-15 | >=16 |
|  |  |  |
| CHD: =<11 | 14-15 | >=16 |
|  |  |  |

Supplementary figure 3: Fitted plot of Dose response meta-analysis, One-stage random-effect dose-response Linear and Cubic spline models. Reference age : 13 year, dash lines(curves):95%CI for the fitted line (curve)

|  |  |
| --- | --- |
|  |  |
|  |  |
